# The Cocaine Habit

**Published:** 1916-08-05

**Authors:** 


### THE COCAINE HABIT.

### Government Action at Last

A REFORM that has long been advocated in the columns of THE HOSPITAL has at last been effected through the medium of an Order in Council placing severe restrictions upon the traffic in cocaine. Of late one has heard a good deal about the "cocaine scandal," by which is meant, of course, the prevalence—the growing prevalence—of the cocaine habit. There is another "cocaine scandal"—namely, the tardiness of the Government in interfering to stop a mischievous traffic that has been steadily growing for the past ten or twenty years, and had already become notorious many years ago. Almost every other civilised country but our own long ago recognised the evil and took steps to cope with it, but up till now the British Government has either ignored the evidence of the existence of a dangerous practice or has been too respectful of the precious liberty of the British public to interfere between it and its vices. Had it not been for the war and the public disclosure of the fact that the Army was tainted with the drug vice we should probably have had to wait another score of years for this reform; indeed, it is quite possible that we should never have witnessed the reform at all.

### VESTED INTERESTS.

The restriction of the cocaine traffic, we may take it, is a direct outcome of the war. It is a piece of emergency legislation effected, as it were, by the stroke of a pen, which in normal times could never have been enacted without an insufferable amount of controversy. All sorts of people have a vested interest in other people's misfortunes, and always we shall find, as we always have found, that we cannot take one step forward without giving offence to someone. One man's undoing is another man's perquisite, and if we were living in peaceful times we should have heard the loud and bitter squealings of the men whose pockets are going to suffer because lives are going to be saved and men are going to be spared from degradation. Even now it would not be surprising to learn that behind the scenes the cocaine traffickers have been doing their best, by all sorts of cunning devices, political influence, and what not, to whittle down the regulations to something quite innocuous. By cocaine traffickers we do not mean those clandestine mongers who waylay soldiers in bars and brothels, for these have no political influence; we mean those highly respectable and well-dressed quacks who out of the fortunes of quackery can hobnob with their betters. These are the men that put the brake on the wheel of progress.

### INEFFECTIVENESS OF THE POISON LAWS.

Now, hitherto the sale of cocaine has not been subject to any sort of effective control. It is true that the Pharmacy Acts prohibit its retail sale except by duly qualified chemists and druggists, and, further, these Acts require that the purchaser shall be known or introduced to the seller, and that the seller shall observe certain regulations as to the labelling of the package, and shall make certain entries in the Poison Book. This sort of mechanical control is practically useless, for who is there who does not know some chemist or other or cannot get an introduction to one? To do justice to the chemists, they have, on the whole, exercised their discretion wisely and have recognised that all that is lawful is not expedient; but this sort of business cannot be left to the variable discretion of the individual; it is neither fair to the individual nor in the public interest that one chemist shall refuse to sell cocaine while his neighbour sells it readily. The Pharmacy Acts, therefore, are useless to cope with this evil, and it redounds to the credit of the Pharmaceutical Society—the body which administers the Acts—that they have not only recognised and acknowledged this, and put no obstacles in the way of the enactment of the new regulations, but have, in the public interest—and, we believe, in the best interests of pharmacy—done all in their power to assist the Government in overcoming obstacles to ensuring a proper control over the sale of habit-forming drugs. This is all in accordance with the highest traditions of a society which, though it has, in the course of its long history, made many deplorable errors of judgment and has, through failure to ascertain facts, committed faults in the government of its domestic affairs, has always endeavoured to act in the best interests of the public.

### THE REGULATIONS EXAMINED.

Let us examine the new regulations. In the first place the importation of cocaine into this country is prohibited except under licence. The effect of this provision will be to enable the authorities to keep the sources of distribution under supervision (it would, perhaps, be better to say the main and legitimate sources); but it will no doubt be found that this regulation will put a premium on smuggling, which, of course, can be checked by the usual means and, better still, by the institution in exporting countries of regulations restricting the export. As regards the distribution of cocaine, the regulations make it an offence for any person to "sell, give, procure, or supply, or offer to sell, give, procure, or supply cocaine to or for any person, other than an authorised person, in the United Kingdom," except under certain well-defined conditions. These conditions are that cocaine may be supplied on the written order of a medical practitioner, who must date and sign the prescription with his full name and address and qualifications, and mark it with the words "Not to be Repeated"; he must also specify the total amount of cocaine to be supplied, "except that where the medicine to be supplied on the prescription is a proprietary medicine, it shall be sufficient to state the amount of the medicine to be supplied." The prescription must be dispensed by a duly qualified person, and must only be dispensed once on the same prescription, except in pursuance of fresh directions duly endorsed. Why the exception should be made in the case of a proprietary medicine it is difficult to understand; if practitioners prescribe proprietary medicines containing cocaine they should surely know the quantity of cocaine, therefore why not state it on the prescription? The regulations also make it an offence for any person other than a medical practitioner, a registered dentist, a veterinary surgeon, a chemist or a firm of chemists, or a person licensed to import cocaine to have the drug in his possession. It will be observed that it is an offence for an unregistered dentist to have cocaine in his possession—a "misfortune" which no one will deplore but the unregistered dentists themselves. To some extent the regulations apply to raw opium, but not to galenical preparations of opium. They do not apply to preparations containing one part of cocaine in a thousand; we think this exception is unfortunate, for we would like to see the drug banned, lock, stock, and barrel, except so far as its legitimate medicinal uses are concerned. Save for the few points to which attention has been drawn, we agree entirely with the new regulations; they are not one bit too drastic, and if properly administered—as no doubt they will be—they should lead to a measurable decrease in a pernicious habit.

